# Health Workforce Deployment, Attrition and Density in East Wollega Zone, Western Ethiopia

**Published:** 2010-03

**Authors:** Yohannes Hailemichael, Challi Jira, Belayneh Girma, Kora Tushune

**Affiliations:** 1 Department of Health Planning and Health Service Management, Jimma University; 2 Addis Continental Institute of Public Health, Addis Ababa

**Keywords:** Decentralization, Health workforce, density, attrition, deployment, West Ethiopia

## Abstract

**Background:**

In East Wollega Zone, despite the success in creating considerable number of health facilities, short-age of health personnel, geographical imbalance and increasing attrition is found to be a persistent barrier to the effectiveness of the health system. However, available data is not rich enough to provide reliable information as to what extent these problems exist in the Zone. Hence, this study was conducted to assess health workforce density, deployment and attrition in East Wollega Zone.

**Methods:**

A six years retrospective record review from 2000–2005 was conducted between February 1, and March 30, 2006 in eleven randomly selected districts of East Wollega Zone. Data obtained from records and interviews made with selected resource persons were organized by triangulating quantitatively and qualitatively. Quantitative data was analyzed using SPSS 12.01 for windows and thematic frame work analysis was used for qualitative data.

**Results:**

Health workforce deployment rate for the years 2000–2005 ranged from 8.2% to 15.4 %. In contrast, attrition rate for the same period ranged from 2.9 % to 8.5 %. Attrition rate for the time after decentralization (2003–2005) was nearly two times greater than before decentralization (OR, 2.04, CI, 1.51, 2.85, P=0.00). Moreover, attrition rate was nearly three times greater for a high level professional when compared to the lower level (OR, 3.15, CI , 2.63, 4.37, P=0.00). Attrition rate for males was two times higher as compared to females (OR, 2.07, CI, 1.67, 3.74, P=0.00). About (26.3%) of all health workers and (36.7%) of nurses and midwives were deployed to the capital town of the zone. Factors identified as most likely cause for the lower deployment and higher attritions were budget related constraints, lack of continuing education opportunity and poor career development.

**Conclusions:**

The number of health personnel in East Wollega was low both by international standards and relative to the national density. Moreover, attrition was higher for the time after decentralization process. Hence, measures that seek to increase the size of the health workforce through increased recruitment, higher retention of existing staff and better geographical balance have to be urgently explored.

## Introduction

The current human resources shortage in the health sector, mainly of African countries threatens the realization of any plan for scaling- up interventions to control the spread of diseases like HIV/AIDS, Malaria and Tuberculosis (1).

Health workforce in Africa has been very low and is unable to match the rapidly growing population and needs. Indeed, Sub-Saharan Africa (SSA) has the lowest ratios of health workers to population in the world (2).

Although, bench marking with regard to what should be an adequate health workers density level is difficult, the World Health Report states 2.3 health workers per 1,000 people is required to attain adequate coverage of essential health interventions and core MDG related health services (3).

The Joint Learning Initiative (2004) estimated the average number of health workers in Sub-Saharan Africa to be about one health worker per 1000 people compared to more than 10 per 1000 people in Norway and Finland (4). Concerning the density of doctors and nurses, the regional differences are enormous (4). Doctors' density ranges from a high of 6 per 1,000 in Italy to a low of 0.022 per 1,000 in Malawi. Nurses and midwives density ranges from 22 per 1,000 people in Finland to only 0.22 nurses and midwives per 1,000 in Ethiopia (3,4,5). According to the report of Federal Ministry of Health Ethiopia has a total health workforce of 55,373 which translates to 0.7 health workforce per 1,000 populations (6).

In Ethiopia, as it is also the case elsewhere in the developing world, shortage of health personnel, geographical imbalances in the number of health workforce and increasing attrition are among the most pressing problems of the health system. Ethiopia has about 0.027 physicians per 1,000 people. This is 3.5 times lower than the SSA average of 0.1 per 1,000 people (5). Health workforce shortages are linked to decreasing student enrollment in health training institutions, failure of employing professionals at the right time and high attrition among those already employed (7).

In the last decade, regardless of continued expansion of output from health worker training schools, available health facilities in rural areas are not good enough to attract, recruit and retain health personnel. Earlier study in Ethiopia documented that attrition rate for doctors between 1997 and 2001 was 15.2 % (5).

Several studies from Africa documented that attrition resulted from one or more attributes of the work environment such as deteriorating living and working conditions, weak performance management, problems pertaining to leadership and supervision structures, inadequate equipment and supplies, lack of recognition for good work, stress due to heavy workload, and limited opportunities for career development and advancement (8–13).

The review of Health Sector Development Program II indicated that, the major challenges in relation to human resource in the sector are poor deployment and poor retention of health professionals; poor human resource management; shortage of budget and irregularity of continuing education (14).

As in many other African countries Ethiopia commenced rapid decentralization in July 2002 with stated goal of improving the efficiency, equity, accessibility, responsiveness, and quality of health service delivery by increasing local authority and introducing flexibility in hiring practices. Nonetheless, human resource management skills are generally weak at the peripheral level for attracting and retaining staff (5).

In East Wollega Zone, despite the success in creating considerable number of health facilities, the human resources achievement remained unimpressive. The work force expected to provide services is deficient both quantitatively and qualitatively. Health facilities, particularly, in rural areas are still challenged by lack of adequate personnel (15).

However, available data are not rich enough to provide reliable information concerning the source of the problems and to what extent these problems exist in the Zone.

Hence, the aim of this study was to assess health workforce density, deployment and attrition in pre and post decentralization period in public health facilities of East Wollega Zone.

## Materials and Methods

To assess the situation of health workforce deployment, attrition and density, a six years retrospective record review from 2000–2005 was conducted between February 1, 2006 and March 30, 2006 in East Wollega Zone which is located at about 331 kms West of Addis Ababa.

The projected population of the Zone for 2005 was estimated to be 1,647,576. The Zone was divided into 21 *woredas* (administrative districts). There were 2 hospitals, 15 health centers, 82 health stations and 107 health posts; all were public. The health service coverage was 80% in 2005(14). The health workforce of the zone comprises of 11 high level professionals, 276 middle level professionals, 316 low level professionals, 136 frontline workers and 380 administrative staff members (15). This paper is part of a study which aimed at assessing health workers perspectives on incentive structure: the case of East wollega zone (16).

The data for this study was obtained from eleven randomly selected districts and a zonal office. Nine hundred twenty six health workers' record was reviewed and 16 interviews were conducted with managers and health workers as key informants in the selected districts. The interview was transcribed and analyzed thematically for the purpose of triangulating the quantitative results. Information on staff deployment and attrition was compiled from personnel document and payroll for the six years. Data were collected by diploma and first degree level professionals using an anonymous pre-tested interview guide, semi-structured questionnaire and check lists for records reviewed. Information on factors influencing deployment and attrition of health workers were the major themes for the in-depth interview.

Data processing and analyses was made using SPSS for windows version 12.1. Frequency and proportions were computed for each item and descriptive statistics were used for description of the data. Comparison was made between different levels of professionals that were 395,539 and 612 at the end of the year 1999, 2002 and 2005, respectively. A deployment and attrition rate were computed in relation to the time before and after decentralization and a P-value of less than 0.05 was considered to be statistically significant. Qualitative data is analyzed thematically and presented in tables. The study was approved by Public Health Faculty Ethical Review Committee of Jimma University. Written and verbal consent was obtained from health managers and participants of this study. Anonymity and confidentiality was assured for all the information provided.

***The following operational definitions were used;***

***Decentralization***: The transfer of authority, responsibility and accountability from the Zonal Health Department to the District Health Office.

***Period before decentralization:*** The three years time (2000 – 2002)

***Period after decentralization:*** The three years time (2003 – 2005)

***Attrition rate**:* Is the number of health workforce who drop out their job in specified period of time as numerator and available health workforce at the end of the same period as denominator (55/539 and 115/612 for time before and after decentralization respectively).

***Deployment rate**:* Is the number of health workers recruited and assigned for the job in specified period of time as numerator and available health workforce at the end of the same period as denominator (199/539 and 188/612 for the time before and after decentralization respectively).

***Density of health workers**:* Number of health workers per 1,000 people.

***High level professionals**:* Include medical specialists and general practitioners.

***Middle level professionals**:* Include first degree and diploma level health professionals.

***Low level professionals**:* Include all certificate level health workers excluding the front line workers.

***Front line health workers**:* Include primary health workers, primary mid-wife and health extension workers.

***Health workforce**:* Is generically used to mean all health cadres (only health professionals) from the primary health care to the zonal level.

## Results

**Figure 1:**
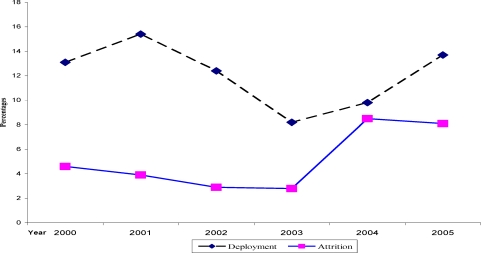
Trends in health workforce deployment and attrition, for the Year 2000 – 2005, East Wollega, February 2006.

A total of 926 personnel documents were reviewed and interview was conducted with 16 key resource persons. The study revealed that during the six years time (2000–2005), about 387 health professionals were deployed and 170 quit their job. Deployment rates for the six years period (2000–2005) ranged from 8.2% to 15.4 %. However, deployment rate was greater for the period before decentralization (36.9% Vs 30.7%). Meanwhile, attrition rate for the six years was in the range of 2.8 % to 8.5%. Thus, attrition rate before decentralization was 10.2% while 18.8% after decentralization (Fig. 1).

**Figure 2:**
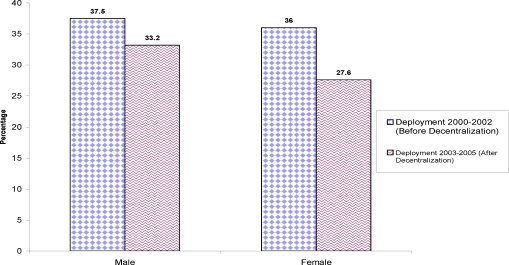
Health workforce deployment by gender, for the Year 2000–2005 East Wollega, February 2006.

Deployment for the time before decentralization was 116 (37.5%) for males and 83 (36.0%) for females while 112 (33.2%) for males and 76 (27.6%) for females after decentralization (Fig. 2).

**Figure 3:**
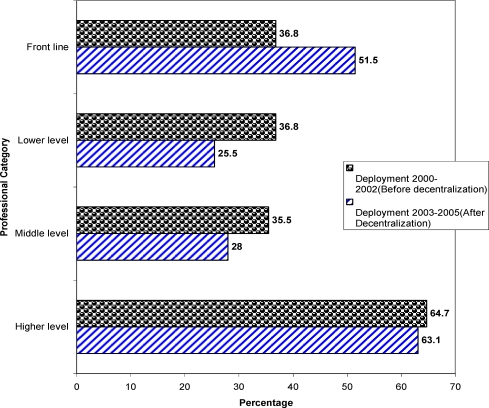
Deployment of health workforce by professional category for the Year 2000–2005 East Wollega

As shown in Figure 3, 11(64.7%) higher level, 103(35.5%) mid level, 71(36.8%) lower level and 14(36.8%) frontline professionals were deployed before decentralization period. On the other hand, after decentralization 12(63.1%) higher level professionals, 91(28%) middle level, 52(25.5%) lower level and 33(51.5%) frontline professionals were deployed.

The attrition rates for professional category revealed that 17 (47.2%) higher level, 78(19.3%) middle level, 67(24.7%) lower level and 8(1.1%) front line professionals left their job. The difference in attrition among professional category was statistically significant (OR=3.15, CI, 2.63, 4.37, P= 0.00). Similarly, data on attrition showed that 55 (10.2%) health workforce during before decentralization and 115 (18.7%) during after decentralization have resigned (OR= 2.04, CI, 1.51, 2.85, P= 0.00) (Table 1). Attrition findings for gender showed that more males, 128 (27.5 %) left their job as compared to females 42 (13.2 %){( OR= 2.07,CI,1.67, 3.74, P=0.00), Table 1}. The density of health workers per 1,000 people was 0.542, while, it was 0.119 for nurses and midwives. However, at Woreda level, the density of health workers per 1,000 people ranged from 0.286 in Guduru to 1.888 in Nekemte (Table 2).

**Table 1:** Attrition of health workforce for the Year 2000–2005 by Profession, Gender and time of decentralization, East Wollega, February, 2006.

		Attrition		
Variables	Yes	No	Adjusted OR	
	N (%)	N (%)	(95%CI)	P-Value
**Profession**				
**Front line**	**8(1.1)**	**64(98.9)**	**1.00**	
**Lower level**	**67(24.7)**	**204(75.3)**	**2.11(1.43,3.23)**	
**Middle level**	**78(19.3)**	**325(80.7)**	**1.90 (1.76,2.42)**	
**Higher level**	**17(47.2)**	**19(52.8)**	**3.15 (1.79,4.21)**	**0.00**
**Gender**				
**Female**	**42(13.2)**	**275(86.8)**	**1.00**	
**Male**	**128(27.5)**	**337(72.5)**	**2.07 (1.51,2.86)**	**0.00**
**Time/Year**				
**2000–2002**	**55(10.2)**	**484(89.8)**	**1.00**	
**2003–2005**	115(18.7)	497(81.3)	2.04 (1.67,3.74)	0.00

**Table 2:** Density of health workforce by district East Wollega Zone, February 2006.

		All health workforce		Nurses and Midwives	
District	Population	Number	Density per 1,000 People	Number	Density per 1,000 People
**Guduru**	**136,239**	**39**	**0.286**	**7**	**0.051**
**Jardega Jarte**	**48,677**	**28**	**0.575**	**5**	**0.102**
**Jimma Arjo**	**83,044**	**35**	**0.421**	**9**	**0.108**
**Abe Dongoro**	**49,293**	**27**	**0.547**	**4**	**0.081**
**Gidda Kiremu**	**147,385**	**63**	**0.427**	**15**	**0.101**
**Guto Wayu**	**135,064**	**39**	**0.288**	**3**	**0.022**
**Jimma Horo**	**89,353**	**61**	**0.682**	**14**	**0.156**
**Ebantu**	**31,517**	**13**	**0.412**	**2**	**0.063**
**Diga**	**57, 842**	**22**	**0.380**	**2**	**0.034**
**Nekemte**	**67,250**	**127**	**1.888**	**39**	**0.580**
**Total**	888,927	482	0.542	106	0.119

According to data obtained from interview of key informants, 13 respondents claimed budget constraints as the cause for decreased deployment rate whereas 10 attributed it to rigid civil service recruitment procedures. Similarly 11 respondents stated that the reason for decreased deployment was poor attention from the Woreda Council while 12 participants perceived the cause to be lack of interest of the prospect employee to work in the remote sites. Fourteen key informants declared that inadequate salary as the reasons for increased attritions followed by 13 respondent who stated the cause as job interference from the external environment (Table 3).

**Table 3:** ‘Attributed’ factors for deployment and attritions rate, East Wollega zone February, 2006

Reasons		Frequency
**Main reasons for decreased deployment (N=16)**		
	Budget constraints	13
	Rigid civil service recruitment procedures	10
	Lack of interest to work in the periphery	12
	Poor attention from the district council	11
**Factors for increased attrition (N=16)**		
	Inadequate salaries	14
	Work interferences from external environment	13
	Lack of remuneration	9
	Lack of opportunities for transfer and promotion	9
	Heavy workload and additional responsibilities	10
	Poor professional development and career	12

* The sum total for the response exceeds 100% because of multiple responses

## Discussion

This study examined the density, deployment and attrition of health workforce for the time before and after decentralization. In fact, despite the wide coverage of decentralization programs and extensive theoretical support, the findings from this study showed that deployment of health workforce is lower during the time after decentralization as compared to the time before it.

Literatures reported that performance of decentralization in many countries is mixed. Van Lerberghe et al. reported that "there is no evidence for an automatic link between decentralization and more effective management of human resources or greater efficiency” (17).

Bossert *et al.* (2000) reviewed decentralization processes in Zambia and Ghana and revealed that decentralization of the health system enhanced governance and accountability (18).

A study in Uganda, indicated that personnel management structures and systems at the district level under decentralization are weak, inadequately staffed, and the district service poorly resourced (19). In the Philippines; negative experiences with decentralization efforts have been more common (20). Decentralization resulted in tighter budgets and an inability of local governments to recruit health workers at the now-higher salaries (20).

In this study, attrition rate for the time after decentralization was two times greater than the time before decentralization. Bond and Dor in a recent paper (2003) argue that the era of structural adjustment, decentralization and free market approaches the loss of health workers through retrenchment and recruitment have deprived poorer and rural communities of access to health services (21).

The findings of lower deployment and high attrition rate after decentralization in this study is similar with the study findings from Columbia, Uganda, Brazil and Zimbabwe with experience of lower deployment rates and high attrition rates in early stage of decentralization reform (22–28).

In this study attrition rate was three times greater for a higher level professional compared to the ones at lower level. One possible explanation for this difference might be the increased demand for higher professional in the private sectors.

Moreover, it was found that Males were twice more likely to quit their job when compared to females (OR, 2.07, CI, 1.51, 2.80, P=0.00). This finding is contradictory with the earlier study from Pakistan that reported higher attrition among females (29). This difference might be explained by the difference in socio-economic and cultural differences of the two populations.

This study identified differences in geographical imbalance in the number of health workforce in different part of the zone. About (26.3%) of all health workforce and (36.7%) of nurses and midwives were deployed to the capital town of the zone where only 8% of the population resided. This finding is similar with the World Bank report (5).

The overall density of health workforce among the Woredas was inequitable and lower than the numbers for Sub-Saharan Africa and Ethiopia (4, 30, 31). The possible explanation for the differences was residential preferences by the health professionals, distribution of health facilities and economic factors. However, the density of nurses and midwives was similar to the density for Afar and Somali regions of Ethiopia (32).

Work interferences from external environment, lack of transfer to urban, lack of training opportunity and the low salary scale push most of the health workforce from the remote sites for seeking better jobs in the urban and private sectors. This finding is similar to the multi-country study that showed refresher training opportunities led to high retention in Zambia, while in Ethiopia a mix of continuing education, provision of housing and establishment of clear career structures improved job satisfaction and retention (8).

In conclusion, the density of health workforce in East Wollega zone was low both by international and national standards. Geographical imbalances measured as differences in the number of health workers per 1,000 people across Woredas are significant. As observed from this study the rate of attrition was higher than normally anticipated. Moreover, lower deployment and higher attritions were closely linked with the time after decentralization. Therefore, implementer of decentralization reform has to initially strengthen the district human resources capacity to make appropriate decisions and to implement them before reform is inaction. Furthermore, strategies that help to increase the size of the health workforce through increased recruitment of new graduates, retention of existing staff and better geographical balance are worth recommended.
